# *Csf2ra* deletion attenuates acute lung injuries induced by intratracheal inoculation of aerosolized ricin in mice

**DOI:** 10.3389/fimmu.2022.900755

**Published:** 2022-09-20

**Authors:** Fuliang Zong, Sha Li, Yifeng Wang, Nan Xiao, Mengyun Deng, Zhipeng Zhang, Duo Su, Bo Gao, Dongsheng Zhou, Lingfei Hu, Huiying Yang

**Affiliations:** ^1^ State Key Laboratory of Pathogen and Biosecurity, Beijing Institute of Microbiology and Epidemiology, Beijing, China; ^2^ Department of Gynecology and Obstetrics, Bethune International Peace Hospital, Shijiazhuang, China; ^3^ Institute of Military Cognition and Brain Sciences, Beijing, China

**Keywords:** RT, *Csf2ra*, ALI, RNA-Seq, GM-CSF receptor, inflammation response

## Abstract

Specific therapeutics are not available for acute lung injury (ALI) induced by ricin toxin (RT). Inhibiting the host immune response in the course of pulmonary ricinosis is hypothesized to be of benefit and can be achieved by impairing granulocyte-macrophage colony-stimulating factor (GM-CSF) signaling, thereby reducing the pro-inflammatory response to exogenous foreign body invasion. However, it is unknown whether mice with impaired GM-CSF signaling can survive after RT inhalation. To test this, colony stimulating factor 2 receptor alpha (*Csf2ra*) knockout (KO) mice that lack GM-CSF signaling and wild-type (WT) mice models of intratracheal exposure to a lethal dose (2× LD_50_) of RT were established. Survival was greater in *Csf2ra* KO mice 21 days after RT inhalation compared with WT mice. Highly co-expressed genes that probably attenuated the pro-inflammatory response in the lung of *Csf2ra* KO mice were identified. Bioinformatics analysis revealed that transcriptome changes involved mostly inflammation-related genes after RT exposure in both *Csf2ra* KO mice and WT mice. However, the activity levels of pro-inflammatory pathways, such as the TNF signaling pathway and NF-κB signaling pathway, in *Csf2ra* KO mice were significantly decreased and the degree of neutrophil chemotaxis and recruitment inhibited after RT-exposure relative to WT mice. RT-qPCR and flow cytometry validated results of RNA-Seq analysis. This work provides potential avenues for host-directed therapeutic applications that can mitigate the severity of ALI-induced by RT.

## Introduction

Acute lung injury (ALI) and acute respiratory distress syndrome (ARDS) are well characterized and readily recognized clinical disorders caused by clinical insults to the lung (1) that are the leading cause of acute respiratory failure, with a high mortality ratio (2). ALI/ARDS can result from direct injury caused by bacterial or viral pneumonia, as well as from toxin inhalation (3, 4).

Ricin toxin (RT) is a 60–65 kDa protein extracted from the seeds of the castor plant (*Ricinus communis*), a widespread plant in tropical regions that is readily available in the global market. RT belongs to the type 2 ribosome-inactivating proteins (RIP) family, is classified as a Category B agent by the U.S. Centers for Disease Control and Prevention (CDC) and is considered a potential bioterror agent due to its high availability and ease of preparation (5). The clinical manifestation of inhaled RT in animal models is closely related to ALI/ARDS, and involves pulmonary proinflammatory cytokine upregulation, massive neutrophil infiltration, and severe edema (5). Pulmonary RT intoxication may affect the transcriptome profile in lung tissues and lead to inflammatory responses that release inflammatory cytokines and activate pro-inflammatory signaling pathways. Currently, no specific therapeutics are available for the treatment of ALI/ARDS induced by RT (5). New therapeutics are urgently needed to effectively treat pulmonary RT toxicity. Emerging evidence suggests that the dramatic inflammatory response induced by RT exposure is the leading cause of mortality (6, 7). Pulmonary ricin toxicity is characterized by a lung pathology associated with a “cytokine storm” (8–10) that is directly caused by an imbalance between pro-inflammatory and anti-inflammatory cytokines (11). The cytokine storm results from a sudden acute increase in circulating levels of several pro-inflammatory cytokines, including IL-6, IL-1, TNF-α and interferon (12). Therefore, applying disease-modifying countermeasures, including inhibiting the host immune response, is a logical approach to treating the course of pulmonary ricinosis.

Granulocyte-macrophage colony-stimulating factor (GM-CSF) is recognized as a differentiation and growth factor for myeloid hematopoietic cells that regulates immune cell proliferation, growth, differentiation, survival, activation and function (13, 14). GM-CSF also bridges innate and adaptive immunity, playing important roles in modulating the immune response against foreign substances. The colony stimulating factor 2 receptor alpha (*Csf2ra*) gene encodes GM-CSF receptor α (CD116) in mice. Defective GM-CSF receptor signaling results in abnormal development and functionality of immune cells and impairment of the host immune response (14).

Our study aimed to look for effective immunotherapy targets for ALI induced by RT-exposure. We thus challenged *Csf2ra* knockout (KO) mice and wild-type (WT) mice with 2× LD_50_ of RT by aerosolized intratracheal inoculation and compared lung tissue pathology and survival in the groups. We also extracted mRNA at various time points post RT-exposure and applied bioinformatic techniques to characterize transcriptome changes. Our results indicate that *Csf2ra* KO mice have improved survival after RT-exposure and transcriptome analysis suggests this was a result of attenuated pro-inflammatory responses. This work provides direction for finding a therapeutic solution to mitigate severity of ALI-induced by RT.

## Materials and methods

### RT preparation

The RT used in this investigation was donated by the Immunology Laboratory of the Beijing Institute of Microbiology and Epidemiology. Extraction and purification methods for RT were done according to published literature (15). Purified RT was stored in a freezer at -80°C until use and all relevant experiments were conducted in a biosafety level-3 laboratory.

### Laboratory animals

Female C57BL/6N WT mice (aged 8–10 weeks) and female *Csf2ra^-/-^* (*Csf2ra* KO) mice (aged 8–10 weeks) were obtained from Cyagen (Guangzhou, China). All experimental mice used were specific-pathogen-free (SPF). Mice were maintained on a 12:12 h light:dark cycle and adaptively raised for 1 week with free access to water and food before starting the experiment. This study was approved by the Institute of Animal Care and Use Committee (IACUC) at the Academy of Military Medical Sciences (AMMS), ethical approval number IACUC-AMMS-2017-031.

### Mouse toxicity studies

Groups of *Csf2ra* KO mice (n = 10) and WT mice (n = 10) were inoculated intratracheally with 2× LD_50_ of RT (approximately 10 μg/kg body weight solution in PBS 50 μl per mouse). The method of intratracheal inoculation followed published literature (16). Briefly, mice were anesthetized with an intraperitoneal injection of 70 mg/kg body weight pentobarbital sodium. Mice were placed in a supine position, the mouth opened, and the tongue gently moved aside using forceps to better cannulate the trachea. The tracheal opening was visualized by inserting a laryngoscope (Huironghe Company, Beijing, China), then a micro sprayer (Huironghe Company) was inserted at the larynx (near the tracheal bifurcation) of the mouse and intratracheal inoculation of aerosol was administered. Mice were then allowed to recover from the anesthetic. The RT-challenged mice were housed in cages within a biosafety level 3 facility and monitored daily for survival.

### Pathological analysis

*Csf2ra* KO and WT mice were sacrificed at 4 h (n = 3), 12 h (n = 5) and 72 h (n = 5) after intratracheal inoculation of RT. Lungs were dissected and hematoxylin-eosin staining (H&E) used to check for morphological changes in the lung tissue of *Csf2ra* KO and WT mice. The lungs of mice in all groups were immersed in a general-purpose tissue fixation solution for ≥48 h, embedded in paraffin, sectioned to a thickness of 4 µm, and then subjected to H&E and photographed. Pathological score was evaluated by two independent pathologists. A semi-quantitative assessment of lung injury was conducted by estimating the following parameters: alveolar septal thickening, perivasculitis, peribronchiolitis, neutrophil infiltration, lymphocytic infiltration, monocyte infiltration, vascular leakage, alveolar edema, hyaline membrane formation, bleeding, bronchial epithelial sloughing/necrosis, and endothelial injury. Each parameter was graded on a scale from 0 to 4, where 0 = normal; 1 = mild; 2 = moderate, 3 = severe, 4 = very severe injury. The total lung inflammation score was expressed as the summed parameter scores.

### RNA extraction, library construction and sequencing

In this experiment, *Csf2ra* KO and WT mice were divided randomly into four groups. The first was a control group that received an intratracheal inoculation of PBS without ricin and was then immediately sacrificed (0 h) *via* CO_2_ anesthetization followed by cervical dislocation and dissection. The remaining three experimental groups received intratracheal inoculation of aerosolized ricin and mice in each group were sacrificed *via* CO_2_ anesthetization followed by cervical dislocation and dissection at 4, 12 and 72 h after inoculation. During dissection, the lungs of mice were removed and lung lobes ground for RNA extraction and transcriptome sequencing.

Total RNA was extracted from the lung tissue using RNAprep Pure Tissue Kit (Tiangen, DP431), according to product instructions. The purity of the sample was determined by NanoPhotometer (IMPLEN, CA, USA). The concentration and integrity of RNA samples were tested using an Agilent 2100 RNA nano 6000 assay kit (Agilent Technologies, CA, USA). All samples were sent to Easyresearch Technology Company in China for sequencing. All cDNA libraries were sequenced using a paired-end strategy on the NovaSeq 6000 S4 platform, using NovaSeq 6000 S4 Reagent kit V1.5.

### Pre-processing of RNA-Seq data

Sequencing data were filtered using a Perl script that: (1) removed reads containing the sequencing adapter; (2) removed reads with >15% low-quality base ratio (base quality ≤5); and (3) removed reads whose unknown base (‘N’ base) ratio was >5%. The resulting clean reads were stored in FASTQ format. Clean reads were mapped to the reference genome using HISAT2 (v2.1.0). Bowtie2 (v2.2.3) was used to align the clean reads to the reference coding gene set.

### Statistical analysis of RNA-Seq data

Statistical analysis and plotting of RNA-Seq data were done using R (R version 4.1.0). Differentially expressed genes (DEGs) were identified using the EdgeR package (17, 18). Selection criteria for identifying DEGs were an adjusted *P*-value <0.05 and a |log_2_FoldChange| >1. The Bioconductor package clusterProfiler (19) for Gene Ontology (GO) and pathway-enrichment analysis (Kyoto Encyclopedia of Genes and Genomes, KEGG) was used to analyze DEGs and gene sets.

### WGCNA

Weighted gene co-expression network analysis (WGCNA) was performed using the online website ImageGP (http://www.ehbio.com/Cloud_Platform/front/#/) with default values. Total expression variance in the top 5000 most variable genes in our study was used as input data to reduce amount of calculation required.

### Time series analysis

The Bioconductor package maSigPro uses a two-step regression strategy for time series analysis. First, significant DEGs among different treatment groups are identified. Second, genes with similar expression patterns are grouped for cluster analysis and visualization. We used the maSigPro package (version 1.62.0) (20, 21) to analyze differences in gene expression patterns in *Csf2ra* KO and WT mice at 0, 4, 12 and 72 h post-inhalation.

### Estimating the abundance of immune cells

The abundance of 10 types of immune cells was estimated using ImmuCellAI (22, 23) (http://bioinfo.life.hust.edu.cn/ImmuCellAI). This tool uses a gene set signature-based method with RNA-Seq data.

### Flow cytometry analysis

*Csf2ra* KO mice and WT mice were sacrificed at 0 h (intratracheal inoculation of PBS) and 12 h (intratracheal inoculation of RT), and the lungs of mice were separated and immersed in tissue digestion solution (1.5 mg/ml collagenase A + 0.4 mg/ml DNase I + 1.5 U/ml dispase II in Hank’s Balanced Salt Solution (HBSS) containing 5% FBS and 10 mM HEPES) for 30 min. Digested tissues were ground, filtered and centrifuged at 3500 rpm for 10 min at 4°C. Red blood cells were lysed in 1 ml of RBC lysing buffer (Hao Yang Biological Products Technology Co., Ltd., Tianjin, China) and then single cell suspensions were filtered through a 40 µm nylon cell filter. Cells were counted by a Countess II FL automatic counter (Thermo Fisher Scientific, Waltham, MA, USA). For flow cytometry analysis, 2 × 10^6^ cells were removed per tube and FcγR blocked for 20 min at 4°C using CD16/CD32 antibody (catalog no. 553141, BD Biosciences, San Jose, CA, USA). Cell suspensions were stained with fluorochrome-conjugated antibodies for 30 min and subjected to flow cytometry analysis using a BD FACSymphony A5 flow cytometer (BD Biosciences, Becton Dickinson, USA). The following fluorophore-conjugated antibodies were used: Fixable Viability Stain 510 (mouse, BD Biosciences, 564406); anti-CD45 (mouse, BD Biosciences, 564279); and anti-Ly6G (mouse, BioLegend, 127641). Data were analyzed with Flowjo version 10 (Ashland, OR, USA).

Flow cytometry data was analyzed using two-way ANOVAs followed by Sidak’s multiple comparisons tests. Statistical significance was defined as *P <*0.05. All data are expressed as mean ± standard deviation (SD) from three biological repeats. Statistical analyses and data plots for these data were done with Prism8 (GraphPad Software).

### RT-qPCR confirmation

To validate RNA-Seq analysis, seven genes from the RNA-Seq analysis were selected for RT-qPCR, ensuring genes chosen were detected at 0, 4 and 12 h after inhalation. RNA was isolated from the tissue using RNAprep Pure Tissue (KitTiangen, DP431) according to the manufacturer’s instructions. RNA (0.8 µg) was used to synthesize cDNA using ReverTra Ace qPCR RT kit (TOYOBO, FSQ-301). The qPCR primers designed for the detection of mouse cDNA are given in **Table 1**. SYBR Green qPCR Master Mix (Servicebio, G3321-15) was used following the manufacturer’s instructions with 7500Fast DX Real-time PCR (Life Technologies Holdings Pte Ltd, T-1000-BYZB/SIN 5375-2013). All primers were verified for the production of a single specific PCR product with a melting curve program.

**Table 1:** Primer sequences used for RT-PCR in this study.

Gene	Forward primer sequence	Reverse primer sequence
β-actin	5’-GGCTGTATTCCCCTCCATCG-3’	5’-CCAGTTGGTAACAATGCCATGT-3’.
Csf2ra	5’-TGCTCTTCTCCACGCTACTG-3’	5’-GGGGTCGAAGGTCAGGTTG-3’
Itgax	5’-CCAAGACATCGTGTTCCTGATT-3’	5’-ACAGCTTTAACAAAGTCCAGCA-3’
F7	5’-TGTAGGGACCAAGCGTACCT-3’	5’-CCACACAGCAATAACCCATTGAT-3’
Atp6v0d2	5’-GGAAGCTGTCAACATTGCAGA-3’	5’-TCACCGTGATCCTTGCAGAAT-3’
Tnf	5’-CCTATGTCTCAGCCTCTTCTCAT-3’	5’-CACTTGGTGGTTTGCTACGA-3’
Il-1β	5’-GGACCCCAAAAGATGAAGGGCTGC-3’	5’-GCTCTTGTTGATGTGCTGCTGCG-3’

### SDS–PAGE and western blotting

The lung of mice was lysed in RIPA lysis buffer (Solarbio, protease inhibitors and phosphatase inhibitors). Equivalent protein quantities were subjected to SDS–PAGE and transferred to nitrocellulose membranes. Membranes were blocked in 5% non-fat milk or 5% BSA for 1 h at room temperature, and then probed with the indicated primary antibodies, followed by the appropriate HRP-conjugated anti-mouse/rabbit secondary antibodies (KPL). Immunoreactive bands were visualized with a chemiluminescence kit (Pierce). Antibodies against the following were used: p65 (1:1,000,CST), p-p65 (1:1,000, CST), IκB (1:1,000, CST), actin (1:5,000, proteintech), p-IκB (1:1,000, CST), TNF (1:1000, Abcam), GAPDH (1:5,000 proteintech).

## Results

### *Csf2ra* KO mice acquire protection from RT-induced mortality and morbidity

**Figure 1:**
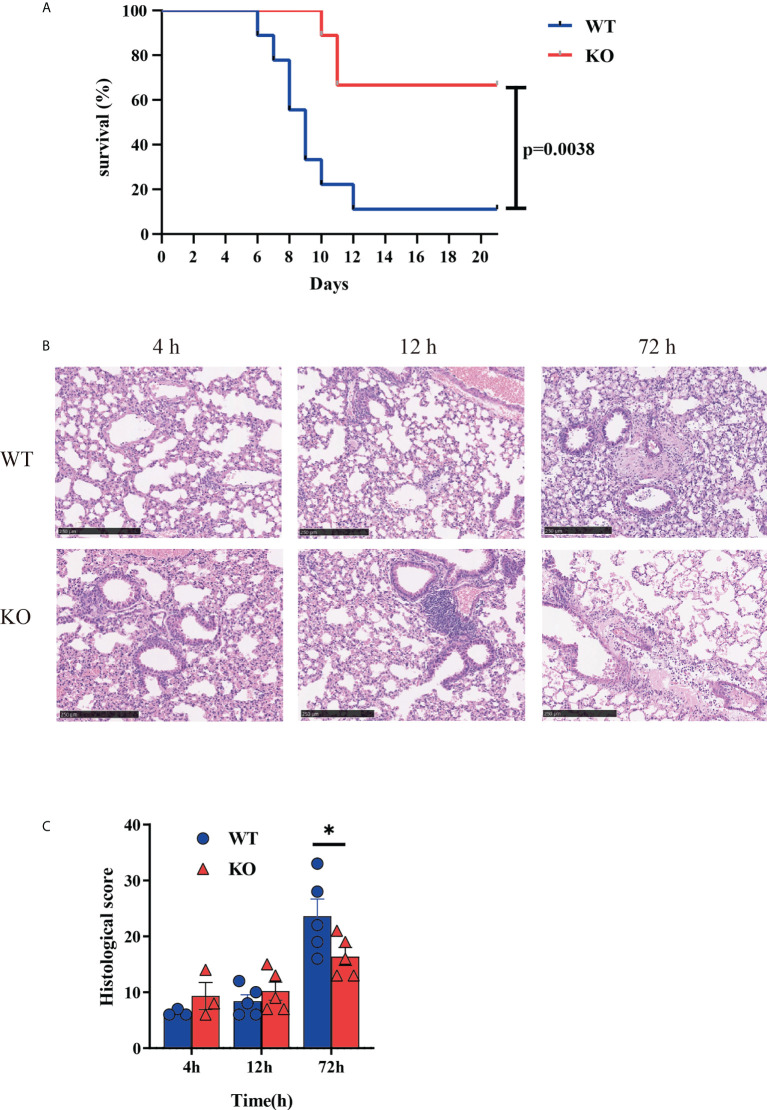
*Csf2ra* KO mice acquire protection from RT-induced mortality and morbidity. **(A)** Survival curves for *Csf2ra* KO mice (n = 10) and WT mice (n = 10); survival rate of the two groups is significantly different (*P* = 0.0038). **(B)** H&E stained lung tissue from each treatment group at three time points. Scale bar, 250 μm. **(C)** Pathological score for each group. *P <0.05.

To investigate the role of *Csf2ra* on the survival time of mice after RT challenge, *Csf2ra* KO (n = 10) and WT (n = 10) mice were challenged with 2 × LD_50_ of RT by aerosolized intratracheal inoculation. We recorded death and survival events for the subsequent 21 days and survival curves are plotted in **Figure 1A**. The *Csf2ra* KO group began to die on day 10 post-RT exposure, while the WT group began to die on day 6 post-RT exposure. On day 21, the survival rates of mice in the *Csf2ra* KO and WT groups were 70% and 10%, respectively. These results indicate that *Csf2ra* deletion decreases mortality in mice after ALI induced by RT.

Given the significantly reduced mortality in *Csf2ra* mice, we next examined whether *Csf2ra* deletion affected pathology of lungs in mice after RT exposure. Histopathological examination of the lung was performed for *Csf2ra* KO mice and WT mice at 0, 12 and 72 h post-exposure. At 72 h post-RT exposure, the lung of WT mice showed increased vascular leakage, the infiltration of inflammatory cells with edema, cellulose, necrosis and sloughing of bronchial epithelium (**Figure 1B**). Lung tissues from KO mice had significantly lower pathology scores than those in the WT group 72 h after RT exposure (**Figure 1C**).

### WGCNA identified gene co-expression networks

**Figure 2:**
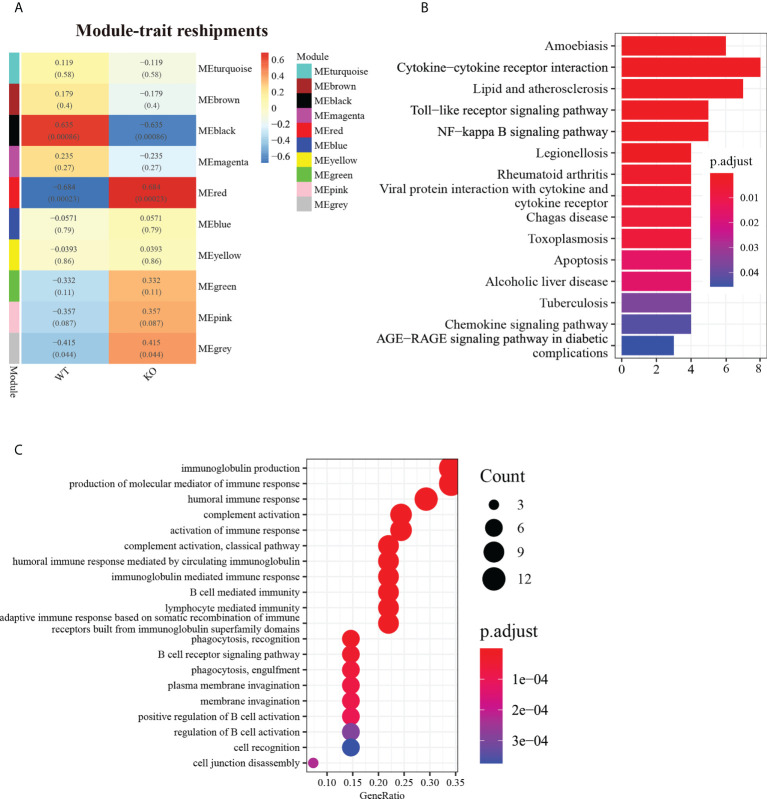
WGCNA identified gene co-expression networks in *Csf2ra* KO mice and WT mice. **(A)** Topological overlap matrix plot showing pairwise gene correlations within each module. Genes within different modules are labeled with different colors according to WGCNA’s conventions. **(B)** The top 15 KEGG entries for genes found in the “black” module. The *x*-axis represents gene count and the *y*-axis is KEGG terms. **(C)** The top 20 entries in GO analysis for biological processes in the “red” module.

A cluster analysis of cleaned RNA-Seq data identified 10 modules (**Figure 2A**). Analysis of the module–trait relationships revealed that module “black” with 48 genes was highly positively correlated with WT mice (*r* = 0.635, P < 0.001), while module “red” with 54 genes was highly positively correlated with *Csf2ra* KO mice (*r* = 0.684, *P* < 0.001). Subsequently, we analyzed the levels of expression of the modules associated with genes in the *Csf2ra* KO mice and WT mice at different time points (**Supplementary Figures 1, 2**). Module “black” included the genes *Tnf, Cd14, Fas, Ccl4, Csf2rb* and *Tnfaip2*. The expression levels of these genes in WT mice were higher than in *Csf2ra* KO mice. While the expression levels of genes in module “red” were lower in WT mice than in *Csf2ra* KO mice. To explore the biological processes in which these co-expressed genes participate, we conducted KEGG analysis and GO analysis. The KEGG analysis showed that the genes in module “black” were related to cytokine−cytokine receptor interaction, the toll−like receptor signaling pathway, the NF−κB signaling pathway and the chemokine signaling pathway (**Figure 2B**). Biological processes in the GO analysis showed that genes in the “red” module were related to immunoglobulin production, complement activation and B cell mediated immunity (**Figure 2C**).

### DEGs analysis revealed that toxic effects induced by RT occurred in both WT mice and *Csf2ra* KO mice

**Figure 3:**
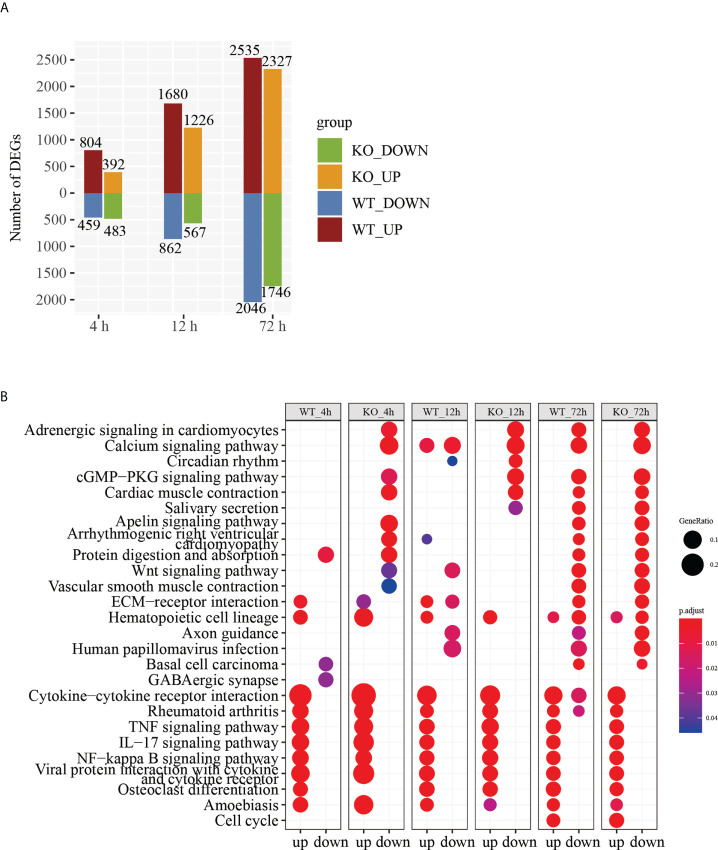
DEGs analysis of RNA-Seq in lung tissue of *Csf2a* KO mice and WT mice after RT-exposure compared to control. **(A)** Bar chart showing the number of DEGs under different experimental conditions. **(B)** The top 25 entries in the KEGG pathway with significant accumulation of up- and downregulated genes in each group under different experimental conditions.

In an effort to make sense of the host response in both the *Csf2ra* KO and WT groups, we compared *Csf2ra* KO mice and WT mice after RT inhalation to their respective steady-states for differential gene expression analysis. A total of 6343 genes were differentially expressed in the *Csf2ra* KO mice and WT mice at three time points (**Supplementary Figure 3**). The number of DEGs in the *Csf2ra* KO mice and WT mice gradually increased over time (**Figure 3A**). KEGG pathway analysis showed that the upregulated genes of each group were related to cytokine-cytokine receptor interaction, the TNF signaling pathway, the IL-17 signaling pathway and the NF-κB signaling pathway in both *Csf2ra* KO mice and WT mice at all time points after RT-exposure (**Figure 3B**). The DEG and KEGG analyses indicate that the lung tissue transcription profiles in the *Csf2ra* KO mice and WT mice were largely dominated by increases in the pro-inflammatory signaling pathway after RT inhalation.

### DEGs analysis of TNF signaling pathway and NF-κB signaling pathway indicate delayed activation decreased in *Csf2ra* KO mice compared to WT mice

**Figure 4:**
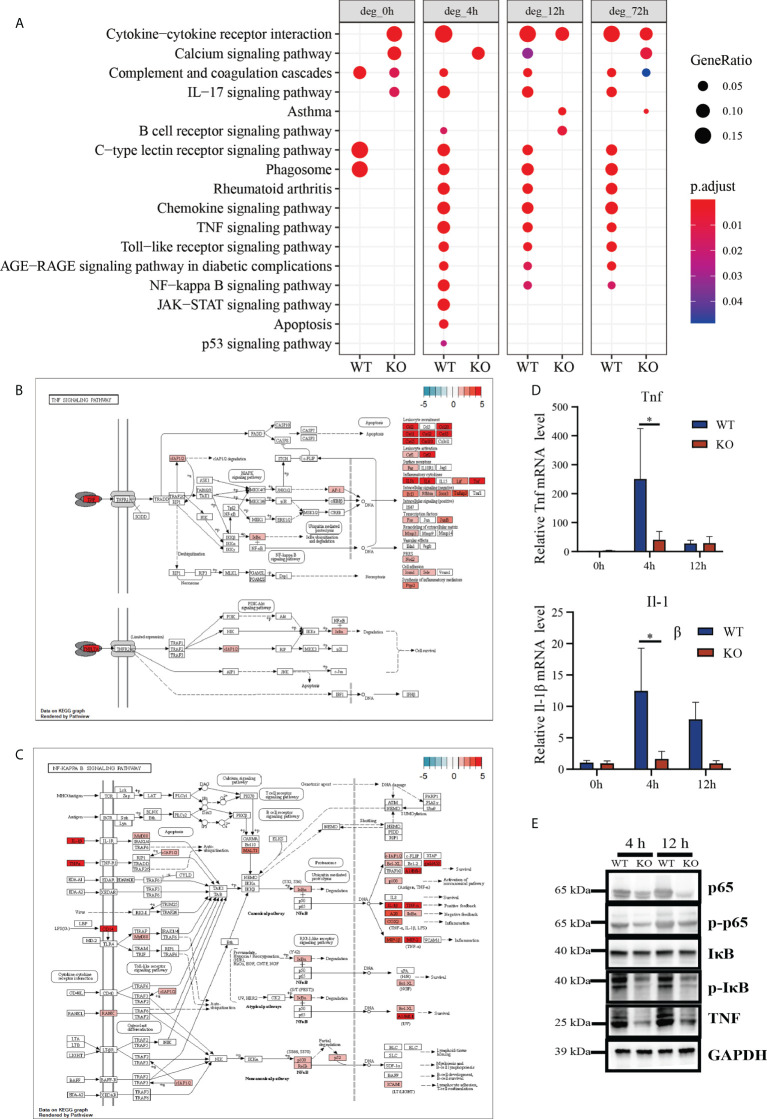
DEGs analysis of RNA-Seq in lung tissue of *Csf2ra* KO mice compared to WT mice at 4, 12 and 72 h post RT-inhalation. **(A)** The top 17 entries in the KEGG pathway of upregulated genes in *Csf2ra* KO mice or WT mice under different experimental conditions. **(B)** The TNF signaling pathway was significantly downregulated in *Csf2ra* KO mice relative to WT mice at 4 h post-RT inhalation. **(C)** The NF-κB pathway was significantly downregulated in *Csf2ra* KO mice relative to WT mice at 4 h post-inhalation. **(D)** RT-qPCR verification of genes identified in transcriptome analysis. Two-way ANOVAs followed by Sidak’s multiple comparison tests were used; *P <0.05. **(E)** Western blot analysis for p-p65, p65, p-IκB, IκB and TNF protein expression levels.

Within-group DEGs analysis revealed pro-inflammatory responses happened in both *Csf2ra* KO mice and WT mice. Next, we analyzed relative pro-inflammatory signal intensity for *Csf2ra* KO mice and WT mice at the same time points. KEGG pathway analysis of the DEGs in the *Csf2ra* KO mice and WT mice indicated that the TNF and NF-κB signaling pathways were significantly downregulated in *Csf2ra* KO mice relative to WT mice at 4, 12 and 72 h post-inhalation (**Figure 4A**).

Having found that DEGs are involved in activating the TNF and NF-κB signaling pathways, we next looked at which genes associated with these pathways were affected by *Csf2ra* deletion using the path-view package in R. Genes in the TNF (**Figure 4B**) and NF-κB (**Figure 4C**) signaling pathways were significantly downregulated in *Csf2ra* KO mice relative to WT mice at 4 and 12 h after RT exposure (**Supplementary Figures 4, 5**). We then chose three essential genes (*Tnf, Il1b*) for validation by RT-qPCR (**Figure 4D**). The protein levels of p-p65, p65, p-IκB, IκB and TNF were estimated using Western blot (**Figure 4E**). *Csf2ra* KO mice decreased p-p65, p-IκB and TNF expression at 4 h and 12 h after RT exposure relative to WT mice. In general, *Csf2ra* deletion in mice results in reduced levels of the TNF and NF-κB signaling pathways both at the transcript level and at the protein level after RT exposure.

### Dynamic gene expression landscape of *Csf2ra* KO mice and WT mice

**Figure 5:**
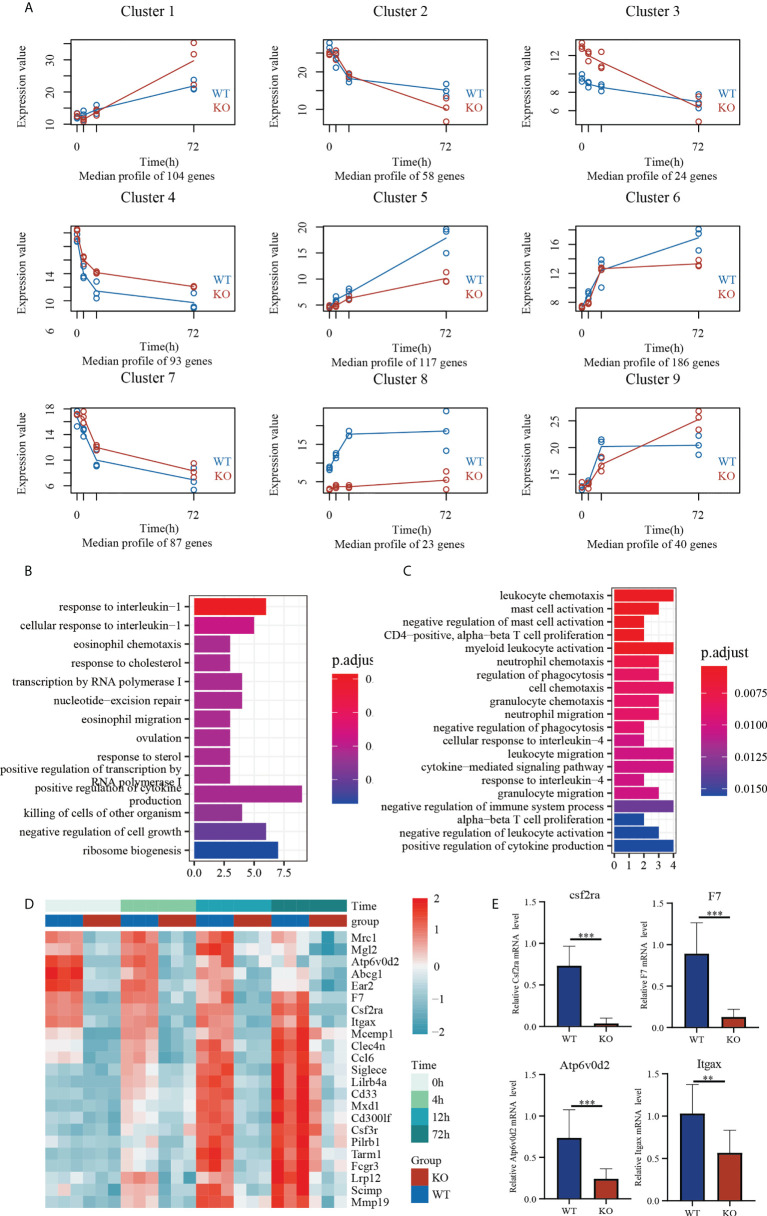
Temporal transcriptome analysis of lung tissues in *Csf2ra* KO mice and WT mice. **(A)** The nine clusters identified by the maSigPro algorithm for the selected genes. **(B)** Analysis of GO biological processes in Cluster 5. **(C)** Analysis of GO biological processes in Cluster 8. **(D)** Heat maps showing expression levels of genes in Cluster 8 in each group. **(E)** RT-qPCR verification of genes with consistently low expression in *Csf2ra* KO mice. Welch’s t test; **P <0.01, ***P <0.0001.

To understand transcriptome changes in the two groups, we used RNA sequencing (RNA-Seq) to document the global expression profiles of each group in a time-dependent manner (**Figure 5A**). For a holistic view, DEGs were clustered based on their temporal expression patterns. Based on the expression patterns of *Csf2ra* KO mice and WT groups, genes were divided into nine clusters; expression differences represent alterations in the transcription levels between groups. For example, cluster 5 had 117 genes and increased expression at 4, 12, and 72 h post-inhalation in both groups. The magnitude of increase in *Csf2ra* KO mice, however, was significantly lower than in WT mice. These genes were enriched for terms such as response to interleukin-1, eosinophil chemotaxis, and positive regulation of cytokine production (**Figure 5B**). Cluster 8 had 23 genes and increased in WT mice sharply at 4 and 12 h post-inhalation, but did not consistently increase in *Csf2ra* KO mice post-inhalation. GO analysis revealed that these gene are involved in leukocyte chemotaxis, neutrophil chemotaxis and the cytokine-mediated signaling pathway (**Figure 5C**). Next, we selected four genes (*Csf2ra*, *F7, Atp6v0d2, Itgax*) that consistently showed low expression in *Csf2ra* KO mice from Cluster 8 (**Figure 5D**) for validation by RT-QPCR (**Figure 5E**). In summary, *Csf2ra* deletion in mice reduced host response after RT-exposure.

### The degree of neutrophil chemotaxis and recruitment in *Csf2ra* KO mice was inhibited compared to WT mice at early time-points

**Figure 6:**
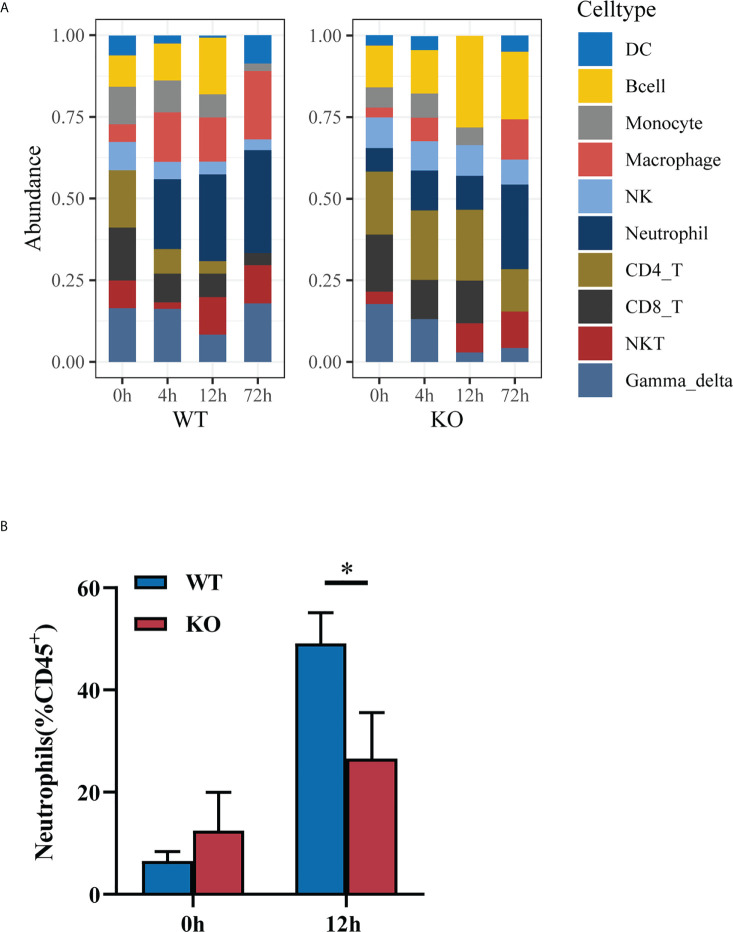
The degree of neutrophil chemotaxis and recruitment in *Csf2ra* ^-/-^ mice was decreased relative to WT mice. **(A)** Immune cell abundance analyzed by ImmuCellAI at different timepoints for *Csf2ra* KO mice and WT mice. **(B)** Bar graph showing the percentage of neutrophils measured by flow cytometry. Two-way ANOVAs followed by Sidak’s multiple comparison tests; *P <0.05.

Given the cytokines and pro-inflammatory signaling pathway alteration between *Csf2ra* KO mice and WT mice after RT exposure, we next investigated whether *Csf2ra* affects the accumulation of immune cells in lung tissue after RT exposure. The relative abundances of 11 types of infiltrating immune cells (TIICs) were quantified with ImmuCellAI in *Csf2ra* KO mice and WT mice (**Figure 6A**). The proportion of neutrophils differed significantly between *Csf2ra* KO mice and WT mice at 4 and 12 h after RT exposure. The proportion of neutrophils in WT mice at 0 h was almost undetectable, while neutrophils were present in *Csf2ra* KO mice at 0 h; this result is consistent with pathological observations in lung tissue. Importantly, the speed of neutrophil chemotaxis and recruitment toward lung tissue appeared inhibited in *Csf2ra* KO mice compared to WT mice.

To test the validity of inferring that neutrophil chemotaxis and recruitment toward lung tissue was inhibited in the *Csf2ra* KO mice at early time-points, we used flow cytometry to identify changes in neutrophil populations during disease progression. The proportion of neutrophils in the lung tissues of WT mice was significantly higher than in *Csf2ra* KO mice at 12 h post-inhalation (**Figure 6B**), confirming the gene set signature‐based method for cell-type composition and that the degree of neutrophil chemotaxis and recruitment was inhibited in *Csf2ra* KO mice compared to WT mice at early time-points.

## Discussion

The therapeutic window for intervention after RT exposure is narrow. The primary treatment strategy is typically supportive care (24, 25). Hence, finding new targets for the treatment of ALI induced by RT inhalation is of major clinical importance. Previous work from our laboratory has shown that expression levels of multiple pro-inflammatory cytokines and transcription factors increased significantly at the early stage of RT-induced ALI (4 h post-exposure) (6). Parajuli et al. (26) demonstrated that GM-CSF priming increased LPS-induced NF-κB nuclear translocation and production of IL-1β, IL-6, TNF-α. De Alessandris et al. (27) confirmed that neutrophilic activation *via* GM-CSFR can play an important role in neutrophilic lung inflammation. Here we show that, GM-CSF signaling deletion attenuated NF-κB and TNF pathway activity following RT challenge in mice. Meanwhile, neutrophil infiltration was significantly reduced in *Csf2ra* KO mice. We speculate blockage of cytokine induction is one of the most direct tactics to blunt the cytokine storm causing ALI/ARDS and lethality.

GM-CSF signaling deletion in mice increases risk for bacterial infection (28, 29). While the exact relationships between RT and GM-CSF signaling deletion remain unclear, the survival data suggest that GM-CSF signal deletion promotes survival in mice after RT exposure. WGCNA was used to identify a distinct co-expression network module associated with host immune response caused by *Csf2ra* KO. Genes in module “black” had the most negatively significant correlation with *Csf2ra* KO mice and genes in module “red” had the most positively significant correlation with *Csf2ra* KO mice. Enrichment analysis indicated that the pathways of genes in the “black” module were linked to cytokine-cytokine receptor interaction, the NF-κB signaling pathway and the chemokine signaling pathway. These genes decrease the likelihood of host immune responses post RT-challenge. Genes in the “red” module were linked to immunoglobulin production, B cell mediated immunity and complement activation. The expression levels of these genes were increased in *Csf2ra* KO mice compared to WT mice, especially in steady states (without RT-exposure). GM-CSF signaling also can act as an anti-inflammatory/regulatory cytokine (30). We speculate that abnormal activation of B cell and complement may partially explain inflammatory cell infiltration in *Csf2ra* KO mice at steady states.

The TNF and the NF-κB signaling pathway were downregulated in *Csf2ra* KO mice compared to WT mice after RT exposure. Tumor necrosis factor (TNF), as a critical cytokine, can induce a wide range of intracellular signal pathways, including apoptosis and cell survival, as well as inflammation and immunity. TNFR1 signaling induces activation of numerous genes that are primarily controlled by two distinct pathways: the NF-κB pathway and the MAPK cascade, or apoptosis and necroptosis. TNFR2 signaling activates the NF-κB pathway, including the PI3K-dependent NF-κB pathway and the JNK pathway promoting cell survival. Different from bacterial or viral infection, the pro-inflammation associated genes in ALI/ARDS induced by toxins have mainly harmful effects. Therefore, the delayed cytokine expression level of genes associated with pro-inflammatory cytokines in *Csf2ra* KO mice after RT-exposure may reduce incidence of ARDS, leading to increased survival.

Another characteristic of pulmonary ricin toxicity is rapid neutrophil infiltration, which ultimately leads to respiratory failure and death. Neutrophils are considered a major hallmark of ALI/ARDS, whether caused by ricin (8–10) or not (31–33). Aggressive or prolonged neutrophil responses result in deleterious inflammatory conditions and tissue destruction. Decreased pulmonary neutrophil infiltration is associated with attenuation of injury severity. In our investigation, the degree of neutrophil infiltration into lung tissue in *Csf2ra* KO mice was much lower than in WT mice. GM-CSF signaling deletion attenuated inflammation and imbalance of the immune system and alleviated severity of ALI induced by RT.

The mitigating effect of *Csf2ra* knockout in mice on RT-mediated lethality may involve multiple aspects. First, the maturation and function of alveolar macrophages (AMs) requires GM-CSF signaling (34). Given their physiological location in lungs, AMs may be the first immune cell to respond to inhaled RT exposure. Moreover, as the time since induction of the RT challenge increased, the number of AMs gradually decreased (35). Thus, AMs may play a central role in the mechanism of ricin toxicity. Korcheva etal. (36) investigated the immunologic properties of AMs *in vitro* and discovered AMs may be the upstream regulators of inflammatory cascades that occur with RT exposure. The *in vivo* results of our study also suggest this. Second, blocking GM-CSF signaling may effectively protect mice from death after RT inhalation by alleviating inflammation. In inflammation, GM-CSF serves as a communication conduit between tissue-invading lymphocytes and myeloid cells (14). A positive feedback loop caused by GM-CSF leads to fast inflammatory cell infiltration and increasing pro-inflammatory cytokine secretion (37, 38). In our research, the lack of GM-CSF signaling in *Csf2ra* KO mice eliminates this pro-inflammatory response, reducing the severity of ALI/ARDS induced by RT. Our study does have limitations. We cannot distinguish between dysfunction of AMs and lack of GM-CSF signaling as the main factor alleviating ALI/ARDS induced by RT. Future studies using conditional knockout mice are needed to explore this question.

In summary, our data revealed that a *Csf2ra* deficiency protects mice from mortality and morbidity induced by RT. The majority of the protective mechanism of ALI/ARDS is *via* reduction in cytokine secretion, decreased activation of pro-inflammatory signaling pathways and decreased neutrophils infiltration. The present study provides a treatment perspective for suppressing the host immune response to protect the host from mortality. This work provides potential therapeutic targets for mitigating the severity of ALI induced by RT.

## Data availability statement

The datasets presented in this study can be found in online repositories. The name of the repository and accession number can be found below: NCBI Gene Expression Omnibus; GSE199606.

## Ethics statement

The animal study was reviewed and approved by Institute of Animal Care and Use Committee.

## Author contributions

FZ: Methodology, Formal analysis, Investigation, Data curation, Writing - Original Draft. sl: Conceptualization, Methodology, Formal analysis, Investigation, Validation. NX: Methodology, Validation. MD: Methodology, Validation. ZZ: Methodology, Validation. YW: Methodology, Formal analysis. DS: Methodology, Formal analysis. BG: Methodology, Formal analysis, Investigation, Resources. DZ: Methodology, Formal analysis, Investigation, Resources. LH: Conceptualization, Investigation, Resources, Data curation, Writing - Review & Editing, Supervision. HY: Conceptualization, Investigation, Resources, Data curation, Writing - Review & Editing, Supervision. All authors have read and agreed to the published version of the manuscript.

## Conflict of interest

The authors declare that the research was conducted in the absence of any commercial or financial relationships that could be construed as a potential conflict of interest.

## Publisher’s note

All claims expressed in this article are solely those of the authors and do not necessarily represent those of their affiliated organizations, or those of the publisher, the editors and the reviewers. Any product that may be evaluated in this article, or claim that may be made by its manufacturer, is not guaranteed or endorsed by the publisher.
