# Supplementary material for: Csf2ra deletion attenuates acute lung injuries induced by intratracheal inoculation of aerosolized ricin in mice
Source: Front Immunol. 2022 Sep 20;13:900755. doi: 10.3389/fimmu.2022.900755 (PMC9531258; doi:10.3389/fimmu.2022.900755)
Supplement: Supplementary file 1 [file DataSheet_1.docx]

Supplementary Material


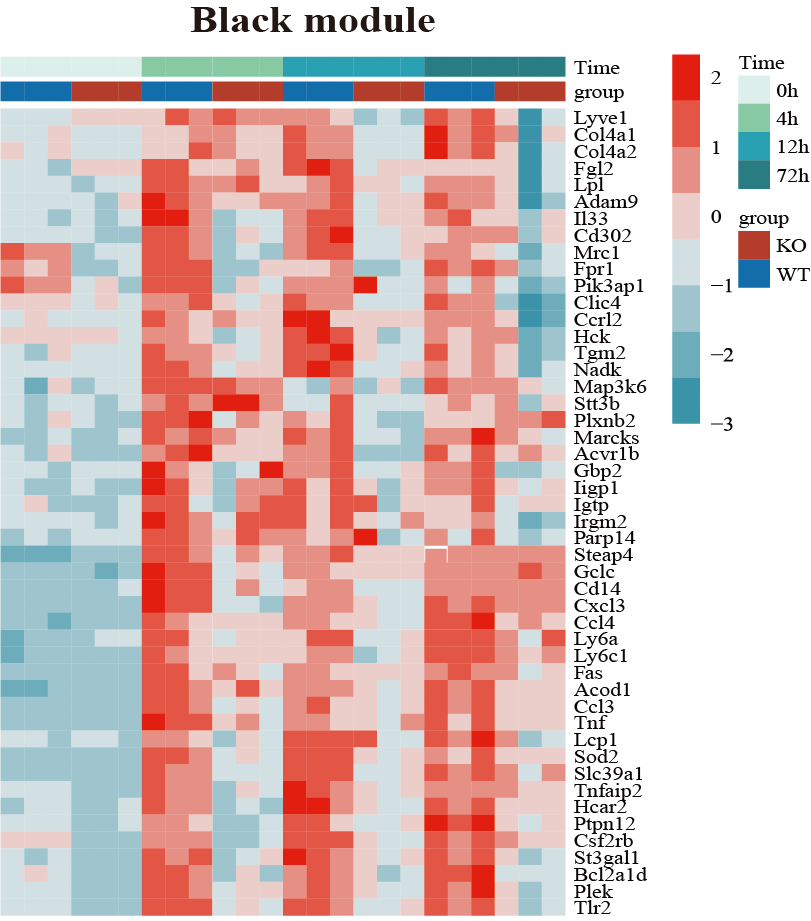


**Supplementary Figure 1.** Expression levels of genes in the “black” module.


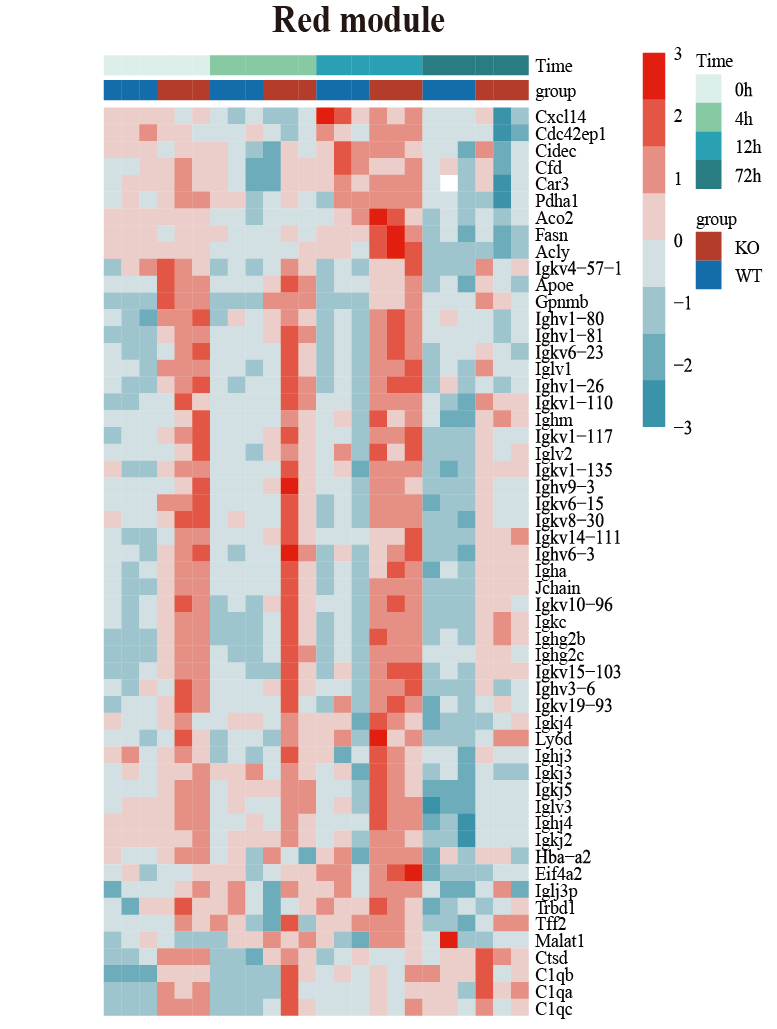


Supplementary Figure 2. Expression levels of genes in the “red” module.


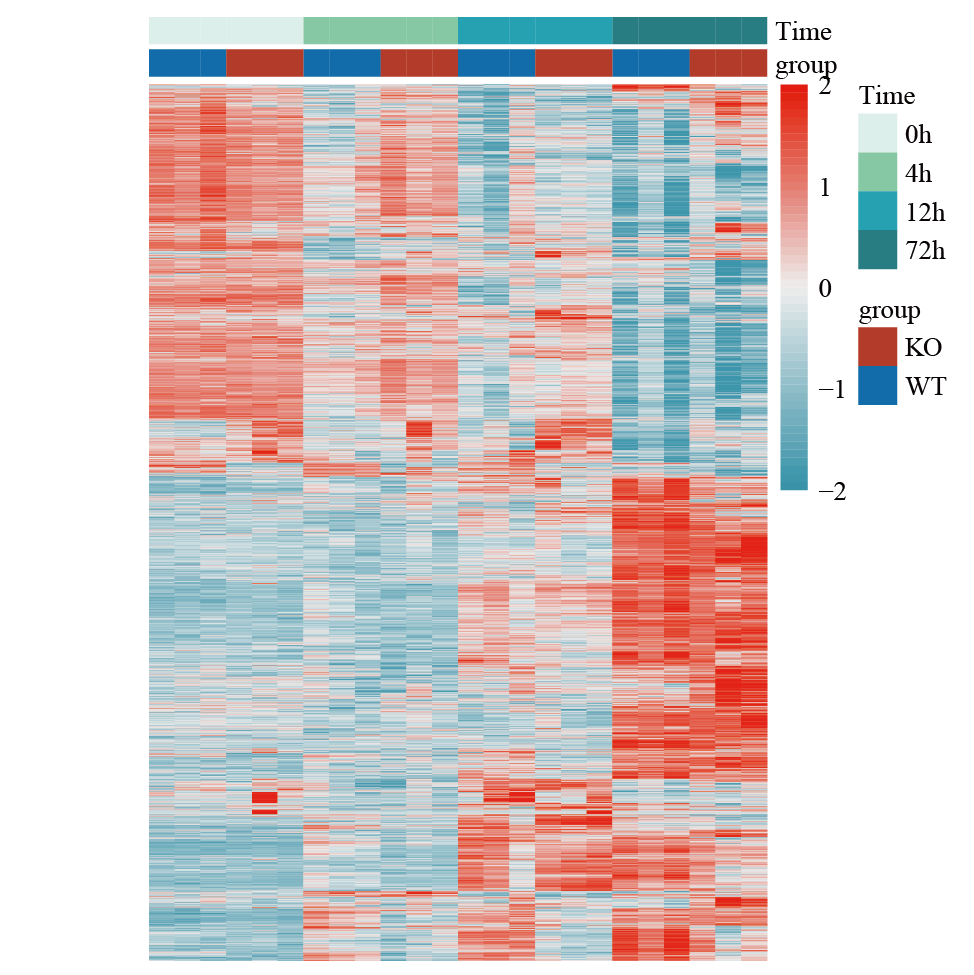


**Supplementary Figure 3.** Heat maps showing gene expression patterns in each group.


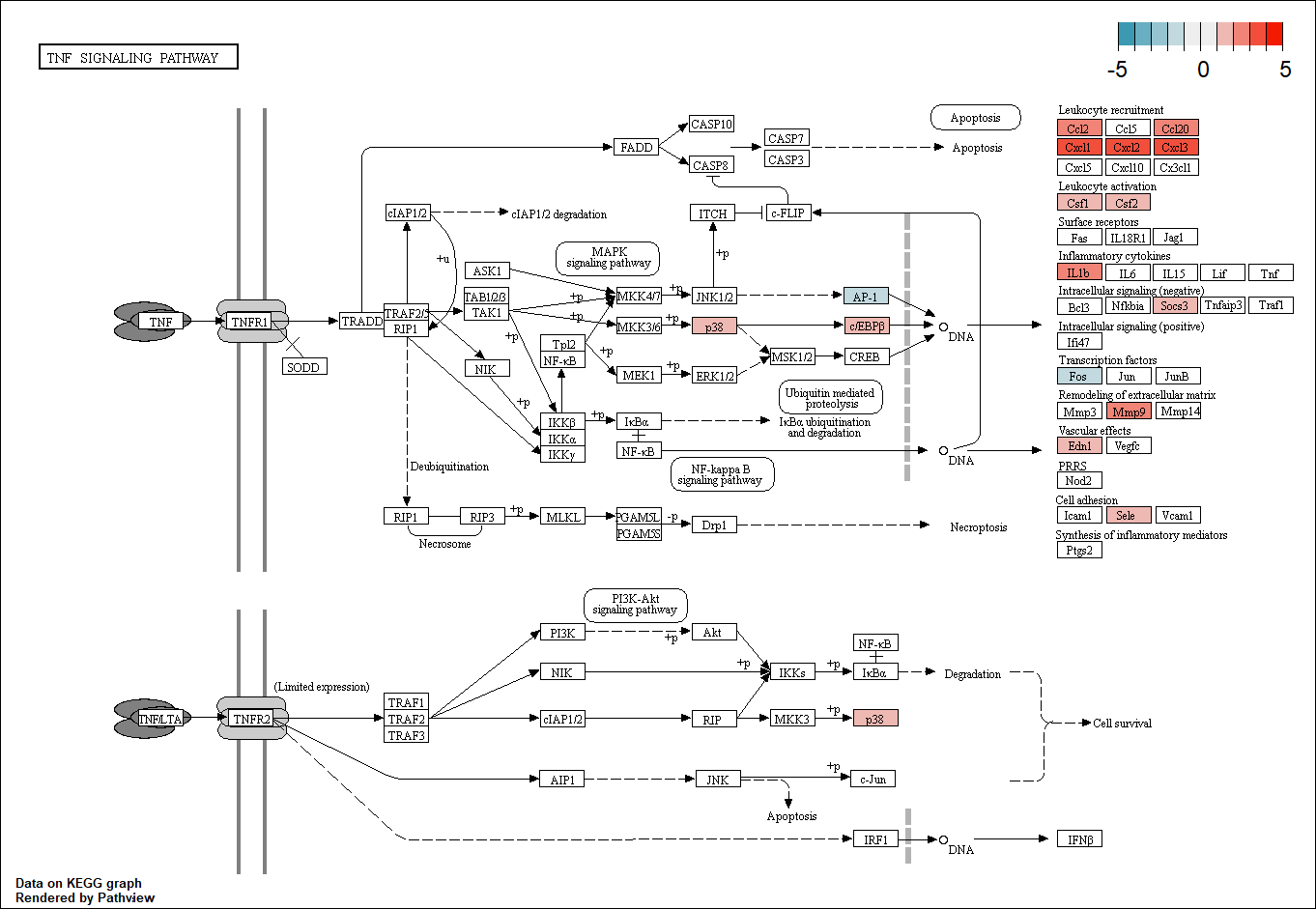


**Supplementary Figure 4.** TNF signaling pathway was significantly downregulated in *Csf2ra* KO mice relative to WT mice at 12 h post-inhalation.


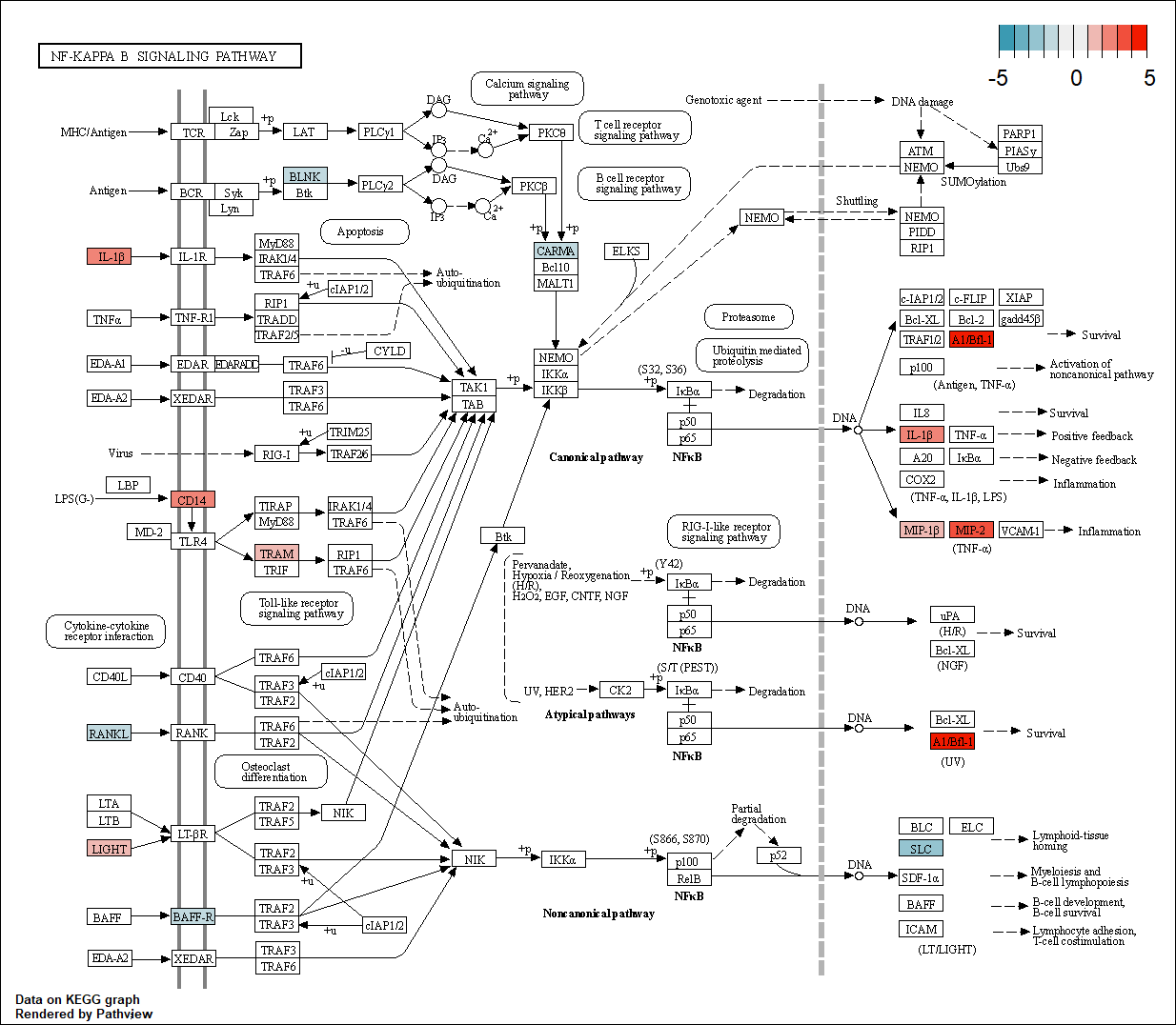


**Supplementary Figure 5.** NF-kappa B pathway was significantly downregulated in *Csf2ra* KO mice relative to WT mice at 12 h post-inhalation.
